# Analyzing the dynamics of social media texts using coherency network analysis: a case study of the tweets with the co-hashtags of #BlackLivesMatter and #StopAsianHate

**DOI:** 10.3389/frma.2023.1239726

**Published:** 2023-10-18

**Authors:** Ke Jiang, Qian Xu

**Affiliations:** School of Communications, Elon University, Elon, NC, United States

**Keywords:** coherency network analysis, semantic network analysis, social media text analysis, hashtag activism, tweet analysis

## Abstract

**Introduction:**

This study examines the associations between time series, termed “coherency,” using spectral analysis. Coherence squared, analogous to the squared correlation coefficient, serves as a metric to quantify the degree of interdependence and co-evolution of individual nodes.

**Methods:**

We utilized spectral analysis to compute coherence squared, unveiling relationships and co-evolution patterns among individual nodes. The resultant matrix of these relationships was subjected to network analysis.

**Results:**

By conducting a case study analyzing tweets associated with the co-hashtags #StopAsianHate and #BlackLivesMatter, we present a novel approach utilizing coherency network analysis to investigate the dynamics of social media text. Frequency domain analysis aided in calculating coherence squared, effectively illustrating the relationships and co-evolution of individual nodes. Furthermore, an analysis of the phase spectrum's slope facilitated the determination of time lag and potential causality direction between highly co-evolved node pairs.

**Discussion:**

Our findings underline the potential of coherency network analysis in comprehending the intricate dynamics of social media text. This approach offers valuable insights into how topics, sentiments, or movements manifest and evolve within the digital realm. Future research should explore diverse datasets and domains to broaden our understanding of this novel analytical technique.

## 1. Introduction

Over the last decade, social media have become an essential component of our everyday lives, resulting in an exponential increase in the volume of data generated through various social media platforms. Researchers have recognized social media texts, including tweets, posts, comments, and reviews, as valuable resources for gaining insights into communication behaviors. Social media texts are diverse in content, ranging from personal updates, news, events, and product reviews. Various factors could influence the content of social media texts, such as user demographics, interests, and social network structure. Users with a similar interest tend to form communities and share similar content. Social media texts are also characterized by their unique structure which includes hashtags, mentions, and emojis. These elements contribute to the context of social media texts and can be used to identify communities and social networks among users. By analyzing the content and structure of social media texts, researchers can identify emerging trends, topics, and sentiment and explore their influence on shaping public opinion, behavior, and preferences (Hou et al., 2021). To enhance our understanding of the dynamics and impact of social media texts, this manuscript introduces a novel approach of using coherency network analysis techniques to examine these texts.

Coherency network analysis is a technique originally used in the field of neuroscience to understand the functional connections between different regions of the brain (Wang et al., 2010). By analyzing the coherence between different brain regions, researchers can identify the functional connections between them and gain insights into how different brain regions work together to perform different cognitive functions (Bowyer, 2016). This technique has been used to study a wide range of cognitive processes, including attention, perception, memory, and language processing (Calhoun et al., 2008; Edmonds, 2020). It can also be applied to understanding the dynamics of social media text and its impact on individuals and society by analyzing the coherence of language patterns within and between different user groups. After extracting language features, such as frequency of words, syntactic structures, and semantic associations from social media texts, this approach uses network analysis to examine the relationships between these features. Through analyzing the coherency of language patterns within and between user groups, researchers can gain insights into how different groups of users communicate and interact with each other.

The increasing availability of real-time data from Twitter, now the X-platform, has made tweet analysis a popular method for studying social media texts. Analyzing tweets allows researchers to gain insights into public opinion, sentiment, and emerging trends (Calabrese et al., 2020; Featherstone et al., 2020; Wang and Zhou, 2021). Twitter has emerged as a prominent platform for digital activism, with hashtags playing a significant role in amplifying the voices of marginalized groups (Tong et al., 2022). Two recent social movements, #BlackLivesMatter and #StopAsianHate, have gained substantial momentum on Twitter. These movements aim to denounce racism, advocate for racial justice, and have garnered widespread attention through their online and offline activist efforts (Lyu et al., 2021; Mir and Zanoni, 2021).

The shared goals of condemning racism and promoting racial justice have fostered cross-racial solidarity and collaboration between the Black and Asian communities (Moon, 2021). Following the tragic 2021 Atlanta Spa shooting, where six Asian women lost their lives, numerous tweets emerged utilizing both hashtags. This unique context provides an opportunity to apply coherence network analysis to explore the interconnectedness of these racial justice movements through social media texts.

Despite representing distinct social movements, #BlackLivesMatter and #StopAsianHate have intersected and gained significant traction due to their shared objectives of combating racism and advocating for racial justice. By examining the coherence network analysis of tweets utilizing these hashtags, we can gain insights into the bridging of these movements and explore the potential synergies and collaborative efforts between the Black and Asian communities in the fight against racism. Therefore, this article presents a case study showcasing the application of coherence network analysis. Specifically, it focuses on examining the dynamics of tweets and the interaction between different Twitter influencers and regular users in shaping the online discourse of the #BlackLivesMatter and #StopAsianHate movements.

## 2. Method: analyzing the dynamics of social media texts via coherency networks

Coherency measures the association between two time series, indicating how well they are correlated (Barnett et al., 2015). Therefore, when analyzing social media texts, it is necessary to calculate time series values that describe the performance of words in the texts. One of the typical values to calculate is the frequency of words over time. To determine the co-evolution of the words used by different Twitter users in a specific period, cross-spectral analyses are conducted among all possible pairs of words to create a coherency matrix of words (Granger and Hatanaka, 1964; Jenkins, 1968; Gottman, 1979). The cross-spectrum is the fourier transform of the cross-covariance function between the series (Bloomfield, 1976). Coherence squared analogous to the squared correlation coefficient is calculated between each pair of words to demonstrate the degree to which word changes (e.g., frequency) are related and how they co-evolve (Gottman, 1979). The slope of the phase spectrum can also be examined to ascertain the time lag to determine potential direction of causality between highly co-evolved pair of nodes. This approach has been used to examine the co-evolutions of international news networks and news frames (Barnett et al., 2015; Barnett and Algara, 2019) and the Trade War discourse on Twitter (Jiang and Xu, 2021).

Given that coherency network analysis generates a matrix of relationships among words, it can be identified as a special form of semantic network analysis. Rooted in the cognitive paradigm and the tradition of frame semantics in linguistics (Fillmore, 1982), semantic network analysis identifies the salience of words and explains the related framing strategies mainly through examining the visibility and co-occurrence of vocabulary in texts. Scholars who have employed semantic network analysis believe that spatial models depicting word relations are indicative of meaning (Barnett and Woelfel, 1988). Recently, semantic network analysis has been applied to analyze social media discourses on various topics, such as healthcare (Kang et al., 2017), natural disasters (Liu, 2012), political campaigns (Eddington, 2018), and social movements (Xiong et al., 2019). In these studies, centrality and cluster analyses were predominantly used to comprehend the framing strategies employed in social media discourses. Similar to other forms of content analysis, it is challenging for this traditional approach of semantic network analysis to reveal the discourse interaction between various types of social media users.

Different from traditional semantic network analysis that focuses on word co-occurrence matrix, coherency network analysis examines co-evolutions of word pairs. It allows scholars to explore how different types of social media actors interact with one another for the co-creation of meaning through collective intelligence (Jiang and Xu, 2021).

In examining social media discourses, previous research has predominantly focused on either the impact of social media actors on a particular topic and their connections within social networks (e.g., Mundt et al., 2018; Larrondo et al., 2019), or the themes and topics that emerge from social media posts (e.g., Ince et al., 2017; Kuo, 2018; Welles and Jackson, 2019; Xiong et al., 2019; Trott, 2021). However, it is important to acknowledge that the actors and content generated through social media posts are inextricably linked. To understand the dynamics of social media text, it is thus necessary to consider both the interactions between social media users and the discourses that arise from these interactions (Wang and Zhou, 2021). Coherency network analysis which incorporates the analysis of time series and cross-covariations, provides a valuable approach to analyze and visualize the complex and dynamic symbolic interactions among the diverse elements of social media texts, including authors, mentions, hashtags, and sentiment.

Specifically, since the coherency analysis produces a matrix of relations, network analysis can be employed to further scrutinize the intricacies and dynamics of social media texts. First, the modularity analysis which gauges the extent to which a network is compartmentalized into clusters, communities or groups (Blondel et al., 2008), can be used to determine the groups of words that have highly co-evolved. The modularity value ranges from 0 to 1, with high modularity networks having dense connections between words within a group but sparse connections between words in different groups (Akbar et al., 2021).

At the word level, betweenness centrality (Newman, 2005) can identify the significant words, such as hashtags, mentions, and sentiment words, that act as bridges or brokers in the coherency network, linking social media conversations emerged from diverse online communities. In addition, eigenvector centrality can be used to examine a word's overall influence in the coherency network (Ruhnau, 2000). On the community/cluster level, weighted degree centrality can be calculated to explore the most central word in each community. The weighted degree value is based on the number of connections for a word, weighted by the coherence squared of each connection (Grandjean, 2015).

Moreover, time lag analysis can be employed to establish the potential causality direction between highly co-evolved word pairs. Specifically, analyzing the slope of the phase spectrum can ascertain the time lag between time series and aid in determining causality direction. A positive or negative slope indicates the extent to which the changes in one series occur before or after the other. To demonstrate how to apply coherency network analysis to the examinations of dynamics of social media discourses on Twitter, this study adopts a case study on the co-evolution of tweets using the co-hashtags of #BlackLivesMatter and #StopAsianHate.

## 3. A case study: co-evolution of tweets with the co-hashtags of #BlackLivesMatter and #StopAsianHate between influencers and regular users

With Twitter becoming a prominent social platform for digital activism, hashtags are used as primary channels to raise awareness of an issue and support a cause (Tombleson and Wolf, 2017). Unlike traditional activism that puts more demand on organizational resources and formation of formal identities to mobilize participation, hashtag activism is non-hierarchical and self-motivated (Bennett and Segerberg, 2012). Through hashtags activism, marginalized groups mobilize online and/or offline actions, raise awareness, demonstrate their needs, and ensure the movements to remain within the public discourse (Simpson, 2018). For example, through #MeToo, women are able to reclaim stigmatized narratives about sexual violence (Gallagher et al., 2019). #OscarsSoWhite successfully brings attention to the Academy of Motion Picture Arts and Science for the lack of diversity (Molina-Guzmán, 2018). #SolidarityisforWhiteWomen reveals the issues with White feminism and enables women of color to address the intersectionality of racial justice and feminism (Kuo, 2018). Two more recent hashtags used in racial justice movements that generated significant social impact are #BlackLivesMatter and #StopAsianHate.

### 3.1. The movements of #BlackLivesMatter and #StopAsianHate

Both Black Live Matter and Stop Asian Hate movements have started and expanded through the use of hashtags (i.e., #BlackLivesMatter and #StopAsianHate). The #BlackLivesMatter movement emerged in 2013 with the purposes of protesting against police brutality and bringing attention to the racism and inequality that the Black community has been facing (Mir and Zanoni, 2021). This hashtag started trending on social media after a series of violent acts by police against Black men in 2014 (Langford and Speight, 2015). After the murders of Breonna Taylor and George Floyd by police in 2020, a new wave of civil unrest staged across the U.S., resulting in an enormous surge of conversations and advocacy for racial equality and justice associated with the #BlackLivesMatter movement. About a year later, the Atlanta Spa shooting led to the death of eight people, with six of whom as Asian women. This shooting is deemed a racial hate crime and one of the many incidents of violence against Asians since the outbreak of the COVID-19 pandemic (Croucher et al., 2020), which brought about the “#StopAsianHate” movement.

Both the movements started or retriggered by murders related to racism and have received nationwide attention. Although there were subthemes unique to each movement (e.g., discontent with police brutality for #BlackLivesMatter and the hate crimes triggered by the COVID-19 pandemic for #StopAsianHate), the discourses of both movements on Twitter shared common themes of motivating participation, connecting online and offline activist activities, expressing emotional responses, and condemning racism (Tong et al., 2022). These common goals are said to fuel Black-Asian solidarity to dismantle white supremacy (Moon, 2021). It is thus not surprising that a lot of tweets posted in 2021 have used the co-hashtags of #BlackLivesMatter and #StopAsianHate, despite of the historical interracial tensions between African-American and Asian communities in the U.S. due to the competition of greater political power and socioeconomic status (Merseth, 2018).

### 3.2. Existing research on #BlackLivesMatter and #StopAsianHate

Although the co-hashtags of #BlackLivesMatter and #StopAsianHate have been widely adopted in tweets after the Atlanta Spa Shootings, there is very limited scholarly research on the dynamics and key social actors in the online discourses using both co-hashtags. Most of the existing research focuses on only one of these movements and the related hashtags. For example, Powell et al. (2023) conducted sentiment analysis over the tweets with the hashtags #BlackLivesMatter and #AllLivesMatter and studied how the adoption of these hashtags affected people's perceptions of the Black Lives Matter movement. In their study, hashtags are considered as communicative tags that signal group identity and provide context for interpreting tweets. Recognizing hashtags as organization instruments to unite fragmented individuals, Chong (2023) studied the key connectors and major themes emerged in the decentralized discourse networks of Black Lives Matter movement. Using a combination of tweets with Black Lives Matter-related hashtags and compiled offline events, Peng et al. (2019) analyzed online crowd behavior dynamics and how it relates to offline events.

Regarding the Stop Asian Hate movement, Lee and Jang (2021) used structural topic modeling and text mining to examine how the Atlanta shooting ignited the public discussions on Twitter, the framing of discourses, and the temporal patterns of topics. Using BERTopic modeling technics, social network analysis, and geo-locational analysis, Chong and Chen (2021) researched top influencers, extracted topics, and identified neighboring hashtags associated with #Chinesevirus and #Chinavirus. Using thematic analysis, Cao et al. (2022) discovered five major themes related to racism against Asian-American and Pacific Islanders (AAPI) in tweets using the hashtag #StopAsianHate: Asian hate is not new; addressing the harm of racism, getting involved in #StopAsianHate; appreciating the AAPI community's culture, history, and contributions; and increasing the visibility of the AAPI community.

Despite there is more and more research examining online social movements through the lens of large Twitter datasets, Chong (2023) called for more attention to studying on why and how individuals and institutions play pivotal roles in the movement and emphasized the importance of adopting a data science approach to study online social movements. Therefore, this study takes a unique angle to study how social media influencers interact with regular users in the discourses using both #BlackLivesMatter and #StopAsianHate through coherency network analysis, which expands the existing research on the dynamics of movements on social media.

### 3.3. Types of influencers on social media

Social media influencers have played significant roles in boosting the visibility and mobilization of social movements through interacting with regular social media users (Hutchinson, 2021). They can be considered as a new type of celebrities who develop a following by sharing content and influence their followers in online social networks (Hendriks et al., 2020). With the increasingly polarized political and social landscape, influencers have been found to maintain their social dominance and become even more influential in setting the discourse on social media (Garibay et al., 2019).

There are different ways to categorize social media influencers (Zulli, 2018). One popular approach is to classify them by the size of followers and divide them into the categories of micro, macro, and mega influencers (Bullock, 2018). *Micro influencers* are defined as those with at least 10,000 but no more than 100 K followers (Vodák et al., 2019). They are considered more accessible and authentic than the other types of influencers. Due to the smaller follower base, they are more likely to engage and build close relationship with their followers (Ruiz-Gomez, 2019). *Macro influencers* refer to those with at least 100 K and up to a million followers (Bullock, 2018) and are considered as the “powerful middle influencers” (Ruiz-Gomez, 2019, p. 16) who are less accessible than the micro influencers but still relatively easier to connect with than mega influencers. *Mega influencers* have the largest follower base among the three. They are defined as those with more than a million followers (Bullock, 2018). They are very well-recognized accounts with authorities in certain topics. Due to the large number, their followers are usually highly diverse in backgrounds and topic interests (Ismail, 2018). Despite of the large reach, mega influencers' engagement with followers could be rather low (Ruiz-Gomez, 2019). Finally, we define those with fewer than 10,000 followers as the *regular* Twitter users in this study.

### 3.4. Research questions

By grouping Twitter users into four categories, this case study examines how different types of Twitter influencers and regular users interact with one another to create and advance the social media discourses about these two movements. It contributes to the existing literature by taking both the actors and the content into consideration when studying the discourse co-evolution network, as well as shedding light on how the co-hashtags are used to connect two racial justice movements to create cross-racial solidarity.

Specifically, we analyze not only the Twitter authors but also their mentioned Twitter handles, the hashtags they adopt, and the sentiment words they use to identify which Twitter users are considered important around certain topics by whom. Moreover, only a limited number of studies have explored the dynamics of social media discourse concerning how diverse types of social media actors collaborate to create meanings through collective intelligence. In addition to examining the distinct contributions of various influencers and regular users to the discourses surrounding two racial justice movements, we delve into the coherency network to uncover the co-evolution of mentions, hashtags, and sentiment words employed by different types of Twitter users.

RQ1: How do different types of Twitter influencers and regular Twitter users, respectively, contribute to the discourses using the co-hashtags of #BlackLivesMatter and #StopAsianHate?
RQ2: How does the discourse from different types of Twitter influencers and regular Twitter users co-evolve over time?

### 3.5. Data and procedure

This study examines the tweets using the co-hashtags of #BlackLivesMatter and #StopAsianHate, published during the 6 months after the Atlanta Spa Shootings on March 16, 2021. In this study, we utilized the Brandwatch platform, a comprehensive social media listening and analytics tool, to capture English tweets containing the co-hashtags #BlackLivesMatter and #StopAsianHate. To ensure a comprehensive dataset, our search query was strategically designed to encompass related hashtags, such as #BLM and #AsianLivesMatter, thereby expanding the sample size and including a diverse array of relevant tweets. Despite the prolific volume of tweets linked to each individual hashtag, the Brandwatch platform yielded a total of 95,025 tweets from 70,795 distinct Twitter users, representing a 100% sample rate. This outcome indicates the platform's successful retrieval of all tweets that met the criteria for containing the specified co-hashtags. Nevertheless, it is essential to acknowledge that the sample size, though smaller in relative terms, warrants consideration in the context of potential API limitations imposed by Brandwatch, which are typically in accordance with Twitter's API guidelines.

After identifying and removing the bots and the tweets created by them, we categorized 45,850 human Twitter users into four groups based on the size of followers: mega influencers with more than a million followers (*n* = 13), macro influencers with 100 K to up to a million followers (*n* = 134), micro influences with a follower base ranging from 10,000 to no more than 100 K (*n* = 1,819), and regular Twitter users with fewer than 10,000 followers (*n* = 43,884).

To examine how different types of Twitter users contributed to the discourse (RQ1), we first analyzed the number of tweets produced by each of them and identified the top authors among each type of Twitter users based on frequency of being retweeted. We then examined the most frequent mentions, hashtags, and sentiment words used by the four types of Twitter users.

To study the discourse co-evolutions among mega, macro, micro influencers, and regular Twitter users (RQ2), we first created a list of words aggregating the most frequent mentions, hashtags, and sentiment words used by four types of Twitter users and then calculated their daily frequencies (185 time points) from the tweets of mega, macro, and micro influencers, as well as regular Twitter users, respectively. The frequencies of these top words used by each type of users at time *t* were correlated with the frequencies of top words not only used by the same type of users but also by the other three types of users at *t* +*1* for the entire time series, creating a series of vectors of correlations, *r*. These vectors were Fourier spectral analyzed, producing a big coherence matrix demonstrating the co-evolutions of top words among four types of Twitter users.

**Figure 1:**
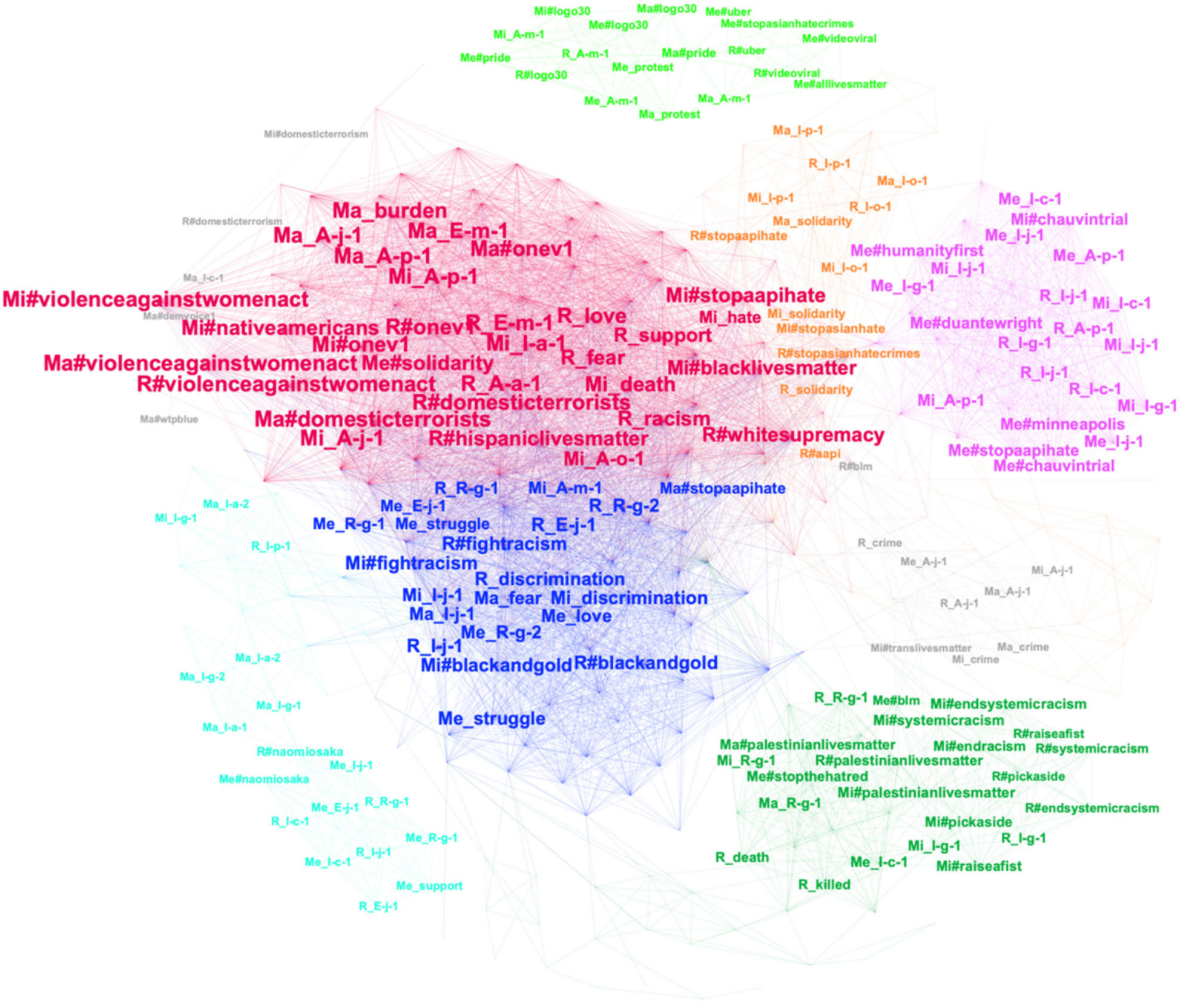
Coherency network of top mentions, hashtags, and sentiment words.

**Figure 2:**
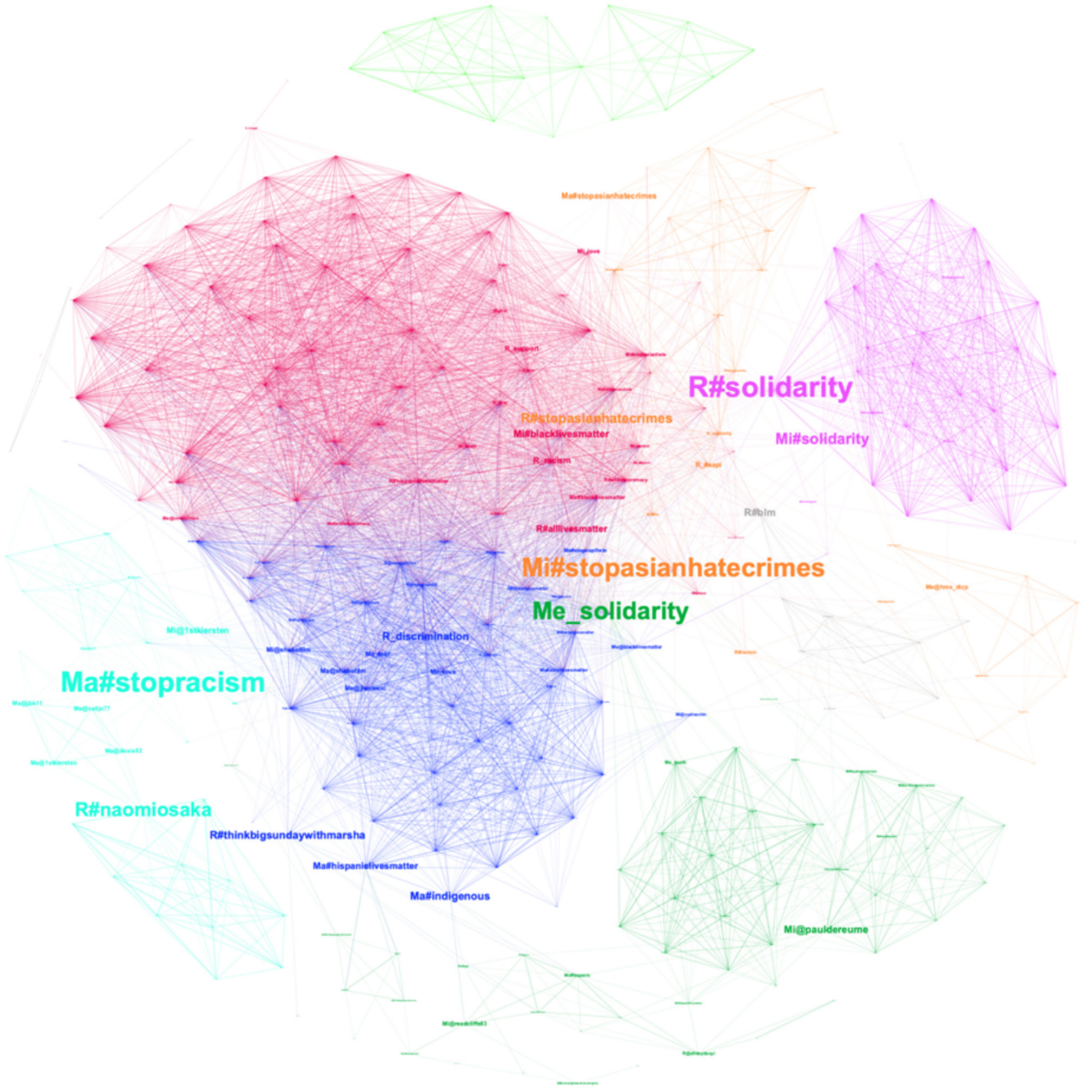
Coherency network of top mentions, hashtags, and sentiment words.

The coherence matrix was examined and visualized (Figures 1, 2) through Gephi (Bastian et al., 2009). Modularity analysis was conducted to explore which set of words highly co-evolved. The coherency score was calculated between each pair of words. Time lag analysis was also conducted to determine the potential direction of causality between the highly co-evolved pair of words. On the network level, the betweenness centrality (Newman, 2005) was used to identify the important nodes (i.e., hashtag, mention, sentiment word) that acted as brokers/bridges in the coherency network. On the cluster level, the weighted degree centrality was calculated to examine the most central node in each cluster. The value of weighted degree was based on the number of connections for a node, but ponderated by the weight (i.e., coherency score) of each connection.

### 3.6. Results

Overall, mega, macro, and micro influencers, and regular Twitter users made different contributions to the discourses with the co-hashtags of #BlackLivesMatter and #StopAsianHate.

#### 3.6.1. Quantity of tweets and retweets

Mega influencer posted the least number of tweets (*n* = 19), but their tweets were mainly original (*n* = 10). In contrast, regular Twitter users produced the greatest number of tweets (*n* = 57,722), but most of them were retweets (*n* = 41,966). From mega to macro influencers, micro influencers, and regular users, the ratios of original tweets to retweets decreased from 1.67 to 0.38, 0.24, and 0.23, respectively (Table 1).

**Table 1:** Quantity of tweets from mega, macro, micro influencers, and regular users.

	**Original**	**Retweet**	**Reply**	**Quote**	**OR**
Mega	10	6	2	1	1.67
Macro	50	130	5	10	0.38
Micro	571	2,415	264	190	0.24
Regular	9,533	41,966	3,244	2,979	0.23

OR refers to the ratio of original tweets to retweets.

#### 3.6.2. Top authors

Among 13 mega influencers, seven were authors, political commentators, cartoonist, chief marketing officer, musician, and comedian, with the rest three as news agencies. The two contradictory tweets about solidarity from an African-American news and political commentator, as well as a New York Times best-selling author, received the greatest number of retweets.

Polarized political views were discovered in the Twitter profile descriptions of macro and micro influencers, as well as regular users. For example, while the most frequent bigrams and hashtags like civil rights, vote blue, #theresistance, and #aapi reflected the left-wing political ideology, Christian conservative, Trump supporter, #maga, #kag demonstrated the right-wing political ideology.

Among the macro influencers, the most retweeted tweets were from an Asian-American journalist. He mainly used the co-hashtags to highlight African-Americans' criminal behaviors and violence against Asian people in the U.S.

For micro influencers, it is interesting that the top two most retweeted tweets also demonstrated opposite attitudes toward solidary between African-Americans and Asian-Americans. Specifically, on March 17, 2021, an Asian-American UI designer used her tweet to ask people not to co-opt the #BlackLivesMatter and #StopAsianHate. Five days later, an Afro-Latino activist, used his tweet to support the co-existence of #StopAsianHate and #BLM.

For regular Twitter users, the most retweeted tweet was a Palestinian dealer of Public Gold, who used the co-hashtags to promote #PalestinianLivesMatter. The second most retweeted tweet was from an African-American company director, who used the co-hashtags to condemn the convict of the Atlanta Spa Shootings. Table 2 lists the top two accounts in each user type and their sample tweets based on the retweet frequency.

**Table 2:** Top two authors by user type and sample tweets.

**Author**	**RT**	**PT**	**Profession**	**Sample tweet**
Mega 1	20,259	3/22/21	Author of NYT bestseller	The #1 violent offenders against black people are other black people. The #1 violent offenders against Asian-Americans are also black people. But both #BlackLivesMatter and #AsianLivesMatterare campaigns dedicated to stomping out white supremacy because, clown world.
Mega 2	152	3/19/21	American news and political commentator, author, and lawyer	Most people don't realize how deep Black and Asian-American solidarity runs throughout US history. Ready to learn? Check my latest 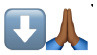 @CNNOpinion #BlackLivesMatter x #StopAsianHate #Solidarity
Macro 1	2,207	5/21/21	Journalist & author of NYT bestseller	An armed black male suspect who was recorded on dashcam video robbing & hitting an elderly Taiwanese Lyft driver in South El Monte, Los Angeles has been arrested. Dandre Powell, 26, is a career criminal with a long history of violence. #BLM #StopAsianHate
Macro 2	1,583	7/23/21	Journalist & author of NYT bestseller	A black woman has been arrested & charged over a spree of antiAsian hate crimes in New York City. Maricia Bell, 25, is accused of assaulting & robbing four Asian victims. #StopAsianHate #BlackLivesMatter
Micro 1	12,916	03/17/21	UI Designer	I see “AsianLivesMatter” trending. Please don't co-opt the #BlackLivesMatter hashtag. Use #StopAsianHate. We must not erase or forget Black oppression in this. Things have been terrible for a long time for many. It is not new. We can lift each other up without co-opting.
Micro 2	807	03/22/21	AfroLatino Activist Artist Antiracist, Doctor	“When BLM was happening this summer…” BLM is still happening. #StopAsianHate and #BLM can coexist. Because anti-Asian racism and anti-Black racism can coexist. Because they always have.
Regular 1	1,240	05/14/21	Dealer of Public Gold	One black American died, the world went #BlackLivesMatter, Two Asians in the United States were shot dead, the world went #AsianLivesMatter Now, more than 100 Palestinians have been killed. #PalestinianLivesMatter
Regular 2	426	03/17/21	Global Finance, Commodities Principal	THIS GUY JUST SHOT 12 AND KILLED 8 ASIAN-AMERICANS and there's not a SCRATCH on him.. he's not Shot to Death from what I can see… I don't see any CHOKE HOLD MARKS or BRUISES… THIS IS SOME BUUUUULLLLL%#@^*^!!!!! #BlackLivesMatter #AsianLivesMatter 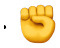 #Atlanta

RT refers to the number of times a tweet being retweeted. PT refers to the posted time.

#### 3.6.3. Top mentions

While mega influencers more frequently mentioned regular users in general, regular users were more likely to mention prominent figures, such as mega journalists, politicians, celebrities, and media outlets. Macro influencers more frequently mentioned other influencers at all levels (i.e., mega, macro, and micro) with the roles of journalists, politicians, celebrities, and media. Micro influencers, in their mentions, displayed a greater inclination toward referencing other micro influencers, along with social activists at both the micro and macro levels. A comprehensive overview of the most frequently mentioned entities by user type is presented in Table 3. The encoding system utilized for representing the top mentions by user types adopts a two-digit format. The first digit corresponds to the type of influencer. The second digit signifies the profession of the mentioned Twitter handles. For instance, “E-j” signifies a mention of a mega journalist. Elaboration regarding the semantic connotations of these alphabetical characters is elucidated within the footnotes of Table 3. It is intriguing to observe that macro and micro influencers, alongside regular users, displayed recurring mentions of individuals who expressed negative sentiments concerning the solidarity between African and Asian-Americans, as identified by Mega 1 and Micro 1 in Table 2.

**Table 3:** The frequency of top mentions by user types.

**Mega**	**F**	**Macro**	**F**	**Micro**	**F**	**Regular**	**F**
R-o	4	E-j	15	I-g	603	E-j	12,251
A-m	3	I-g	14	E-j	237	I-g	6,649
I-c	3	I-j	14	I-a	227	A-j	3,406
I-g	3	A-j	8	E-p	124	I-p	692
I-j	3	E-p	8	A-j	74	I-a	662
R-g	3	E-m	7	I-c	65	E-c	647
E-j	2	I-a	7	A-a	58	E-m	538
A-j	1	A-o	5	A-p	58	R-g	537
A-p	1	A-p	5	R-g	57	E-p	500
		I-p	5			A-p	363
		A-a	4			I-o	321
		A-m	4			A-c	250
		I-c	4			I-c	228
		A-c	2				
		E-c	2				
		E-o	2				
		I-m	2				
		I-o	2				
		R-g	2				

In respect to the initial digit, “Me” designates mega influencers, “Ma” corresponds to macro influencers, “Mi” pertains to micro influencers, and “R” signifies regular authors. As for the second digit, “a” denotes activists, “c” represents celebrities, “j” signifies journalists, “m” stands for media, “o” refers to organizations, “p” indicates politicians, and “g” represents grassroots entities.

#### 3.6.4. Top hashtags

Besides the hashtags of #Black Lives Matter and #Stop Asian Hate, #solidarity was a unique hashtag proposed by mega influencers. Macro influencers expanded these two racial movements to a wider scale by emphasizing hashtags like #indegenours, #allivesmatter, #hispaniclivesmatter, #latinolivesmatter, and #palestinianlivesmatter. Micro influences emphasized more on the hashtags promoting left-wing politics, such as #demvoice1 and #wtpblue. The LGBTQ-related hashtags (e.g., #lgbtq, #transrightsarehumanrights, #translivesmatter) have been used most frequently by regular users. See Table 4 for the top 20 most frequent hashtags by user type.

**Table 4:** Top 20 hashtags used by mega, macro, and micro influencers, and regular users.

**Mega**	**F**	**Macro**	**F**	**Micro**	**F**	**Nano**	**F**
#blacklivesmatter	17	#blacklivesmatter	151	#blacklivesmatter	2,581	#blacklivesmatter	47,952
#stopasianhate	17	#stopasianhate	139	#stopasianhate	2,316	#stopasianhate	36,289
#asianlivesmatter	3	#asianlivesmatter	54	#asianlivesmatter	883	#asianlivesmatter	21,138
#blm	2	#blm	48	#blm	872	#blm	12,672
#georgefloyd	2	#whitesupremacy	13	#stopasianhatecrimes	356	#stopasianhatecrimes	3,622
#alllivesmatter	1	#stopasianhatecrimes	12	#endracism	180	#stopaapihate	1,779
#chauvintrial	1	#stopaapihate	9	#demvoice1	169	#lgbtq	1,057
#duantewright	1	#indigenous	8	#systemicracism	145	#alllivesmatter	917
#humanityfirst	1	#alllivesmatter	5	#endsystemicracism	144	#racism	895
#logo30	1	#domesticterrorists	5	#wtpblue	124	#endracism	876
#minneapolis	1	#aapi	4	#strongertogether	123	#palestinianlivesmatter	876
#naomiosaka	1	#hispaniclivesmatter	4	#lgbtq	107	#georgefloyd	850
#pride	1	#latinolivesmatter	4	#pickaside	107	#stopracism	775
#solidarity	1	#palestinianlivesmatter	4	#raiseafist	107	#transrightsarehumanrights	696
#stopaapihate	1	#thinkbigsundaywithmarsha	4	#stopaapihate	102	#strongertogether	674
#stopasianhatecrimes	1	#violenceagainstwomenact	4	#pride	74	#equality	661
#stopthehatred	1	#blackandgold	3	#fbr	69	#love	661
#uber	1	#domesticterrorism	3	#nativeamericans	64	#demvoice1	589
#videoviral	1	#endracism	3	#racism	64	#translivesmatter	579
		#fightracism	3	#onev1	63	#endsystemicracism	566
		#hispanic	3				
		#love	3				
		#peace	3				
		#pride	3				

#### 3.6.5. Top sentiment words

The sentiment words were determined from the Bing sentiment lexicon (Liu, 2012) using the R programing language. *Solidarity, support*, and *love* were the most frequent positive words, while *violent, racism, hate, crime*, and *killed* were the most frequent negative words used by all four types of users. Macro and micro influencers used the word *discriminatio*n more frequently, while macro influencers and regular user used the word *death* more frequently than the other types of users. *Fear* was more frequently found in micro influencers' tweets, while *oppression* appeared more frequently in regular users' tweets. See Table 5 for the top 10 most frequent sentiment words by user type.

**Table 5:** Top 10 sentiment words used by mega, macro, micro influencers, and regular users.

**Mega**	**S**	**F**	**Macro**	**S**	**F**	**Micro**	**S**	**F**	**Regu**	**S**	**F**
Racism	N	7	Violent	N	30	Violent	N	485	Violent	N	24,606
Solidarity	P	3	Racism	N	26	Racism	N	359	Oppression	N	6,764
Biases	N	2	Solidarity	P	14	Hate	N	273	Racism	N	5,191
Hate	N	2	Support	P	13	Oppression	N	118	Hate	N	3,974
Violent	N	2	Hate	N	10	Support	P	103	Crime	N	1,959
Crime	N	1	Oppression	N	10	Solidarity	P	93	Support	P	1,862
Killed	N	1	Death	N	9	Fear	N	83	Death	N	1,521
Love	P	1	Love	P	7	Love	P	69	Killed	N	1,336
Protest	N	1	Crime	N	6	Crime	N	63	Solidarity	P	1,174
Struggle	N	1	Killed	N	6	Discrimination	N	39	Love	P	929
Support	P	1	Discrimination	N	5	Burden	N	38	Burden	N	613

S refers to Sentiment; N represents negative; P represents positive.

#### 3.6.6. Discourse co-evolutions among four types of twitter users

The discourse co-evolutions among the four types of Twitter users were examined through the coherency network of top mentions, hashtags, and sentiment words. Figure 1 presents the visualization of the coherency network, wherein color-coding is utilized to signify distinct clusters resulting from the modularity analysis. The size of nodes is determined by their weighted degree centrality. The minimum link weight (i.e., coherency score) has been set to 0.5. By setting this threshold, we aimed to emphasize and clearly demonstrate the nodes that exhibit a high level of co-evolution. Nodes with a coherency score of 0.5 or higher indicate a strong association and interconnectedness in the coherency network. Table 6 provides a listing of the hub node (i.e., H) within each cluster based on their weighted degree centralities (WD). Additionally, the table includes the top 10 nodes that co-evolve with the cluster hub based on the coherency score (CO), which varies between 0 and 1.

**Table 6:** Top co-evolutions of the most central node in each cluster of the coherency network.

**Red 21.74%**	**H: Ma#domesticterrorists**	**CO**	**STL**	**Blue 16.46%**	**H: R_E-j-1**	**CO**	**STL**
	**(WD** = **69)**				**(WD** = **67.32)**		
	Ma_burden	0.986	0		Mi#fightracism	0.992	0, −1
	Ma_A-j-1	0.986	0		Mi_discrimination	0.99	0, −1
	Ma_E-m-1	0.986	0		R_discrimination	0.988	0, −1
	Ma#onev1	0.986	0		R#fightracism	0.982	0, −1
	R_E-m-1	0.98	0		R_R-g-1	0.979	1, 0
	R_A-a-1	0.966	0		R_R-g-2	0.975	0, 1
	R#domesticterrorists	0.96	0		Me_R-g-2	0.963	0, 1
	Mi#violenceagainstwomenact	0.955	0		Me_love	0.963	0, 1
	Mi#nativeamericans	0.94	0		Ma_fear	0.963	0, 1
	R#onev1	0.939	0		Ma_I-j-1	0.963	0, 1
	Mi#onev1	0.939	0		Mi_I-j-1	0.963	0, 1
	R#violenceagainstwomenact	0.936	0		Mi#blackandgold	0.944	−1, −2
	R#hispaniclivesmatter	0.925	0, −1		Mi_A-m-1	0.94	0, 1
	Ma#violenceagainstwomenact	0.922	0		R_I-j-1	0.919	0, −1
	Mi_death	0.922	1		R#blackandgold	0.904	−1, −2
	Mi_A-p-1	0.92	0		Me#solidarity	0.901	2, 3
	Mi_A-j-1	0.91	0, −1		Me_R-g-1	0.888	1, 2
	Mi_I-a-1	0.91	0		Me_E-j-1	0.888	1, 2
	R_fear	0.901	0, −3		Me_struggle	0.888	1, 2
	Ma_A-p-1	0.9	0		Me_violent	0.873	−1, 0
**Green 16.46%**	**H: Mi#palestinianlivesmatter**	**CO**	**STL**	**Purple 15.84%**	**H: R_I-g-1**	**CO**	**STL**
	**(WD** = **18.76)**				**(WD** = **27.65)**		
	Mi_I-g-1	0.943	2, 1		R_I-j-1	0.999	0
	Mi#endsystemicracism	0.937	0		R_I-c-1	0.999	0
	Mi#systemicracism	0.927	0		R_I-j-1	0.999	0
	R_I-g-1	0.886	0		R_A-p-1	0.997	0
	Mi#pickaside	0.856	0, 1		Me_A-p-1	0.993	0
	Mi#raiseafist	0.856	0, 1		Me_I-g-1	0.993	0
	R#endsystemicracism	0.791	1, 0		Me_I-j-1	0.993	0
	Mi#endracism	0.729	0, 1		Me_I-c-1	0.993	0
	R#palestinianlivesmatter	0.725	0		Me_I-j-1	0.993	0
	R_R-g-1	0.724	0		Me#chauvintrial	0.993	0
	Me_I-c-1	0.713	0		Me#duantewright	0.993	0
	Me#stopthehatred	0.713	0		Me#humanityfirst	0.993	0
	Ma_R-g-1	0.713	0		Me#minneapolis	0.993	0
	Ma#palestinianlivesmatter	0.713	0		Me#stopaapihate	0.993	0
	Mi_R-g-1	0.713	0		Mi_A-p-1	0.993	0
	R_death	0.7	0		Mi_I-g-1	0.993	0
	R#pickaside	0.66	0, 1		Mi_I-j-1	0.993	0
	R#raiseafist	0.659	0, 1		Mi_I-c-1	0.993	0
	R_killed	0.658	0		Mi_I-j-1	0.993	0
	R#systemicracism	0.658	0, 1		Mi#chauvintrial	0.993	0
**Orange 10.25%**	**H: R#stopaapihate**	**CO**	**STL**	**LB 9.01%**	**H: R#naomiosaka**	**CO**	**STL**
	**(WD** = **25.85)**				**(WD** = **10.26)**		
	R_I-p-1	0.921	−1, 0		Ma_I-a-1	0.787	−1
	Ma_I-o-1	0.913	0		Ma_I-a-2	0.787	−1
	Mi_I-o-1	0.913	0		Ma_I-g-1	0.787	−1
	R_I-o-1	0.912	0		Ma_I-g-2	0.787	−1
	Ma_I-p-1	0.911	0		R_R-g-1	0.748	0, −1
	Mi#stopaapihate	0.906	0		Me_I-c-1	0.717	−1, 0
	R#stopasianhatecrimes	0.898	0, 1		Me_I-j-1	0.717	−1, 0
	R_solidarity	0.883	−1, 0		Me_R-g-1	0.717	−1, 0
	Mi_solidarity	0.883	−1, 0		Me_E-j-1	0.717	−1, 0
	Mi_I-p-1	0.796	0		Me#naomiosaka	0.717	−1, 0
	R#whitesupremacy	0.788	1, 0		Me_support	0.717	−1, 0
	Ma#stopaapihate	0.746	−4, 6		R_I-c-1	0.717	−1, 0
	Ma_solidarity	0.745	0, 6		R_I-j-1	0.717	−1, 0
	Mi_hate	0.743	0, 1		R_E-j-1	0.634	−1, 0
	Mi_A-o-1	0.721	5, 0				
	Mi#stopasianhate	0.717	0, 1				
	R#aapi	0.673	0, 4				
	Mi#blacklivesmatter	0.67	4, 0				
	R_racism	0.669	4, 5				
	R_love	0.661	0, 1				
**LG 5.59%**	**H: Ma#pride**	**CO**	**STL**	**Gray 1 3.11%**	**H: Me_A-j-1**	**CO**	**STL**
	**(WD** = **13.05)**				**(WD** = **7.41)**		
	Ma#logo30	0.881	0		Ma_A-j-1	0.954	0, 1
	Ma_protest	0.881	0		R_crime	0.94	0
	Ma_A-m-1	0.858	0		Ma_crime	0.893	0
	R_A-m-1	0.835	0, 1		R_A-j-1	0.818	0, 1
	R#logo30	0.835	0, 1		R#blm	0.763	0, 1
	Me_A-m-1	0.764	1, 0		Mi#translivesmatter	0.683	−1, 1
	Me#logo30	0.764	1, 0		Mi_crime	0.68	0
	Me#pride	0.764	1, 0		Mi_A-j-1	0.668	0, 1
	Me_protest	0.764	1, 0		R_support	0.514	1
	Mi_A-m-1	0.764	1, 0		Me#blm	0.506	0, 7
	Mi#logo30	0.764	1, 0	**Gray 2 0.93%**	**H: Ma#demvoice1**	**CO**	**STL**
	Me#alllivesmatter	0.719	1		***(WD** **=** **2)***		
	Me#stopasianhatecrimes	0.719	1		Ma#wtpblue	1	0
	Me#uber	0.719	1		Ma_I-c-1	1	0
	Me#videoviral	0.719	1	**Gray 1 0.62%**	**H: Mi#domesticterrorism**	**CO**	**STL**
	R#videoviral	0.719	1		***(WD** **=** **0.93)***		
	R#uber	0.584	1		R#domesticterrorism	0.93	0, 1

For the first digit, “Me” signifies mega influencers, “Ma” represents macro influencers, “Mi” denotes micro influencers, and “R” indicates regular authors. For the second digit, “E” is indicative of mega influencers, “A” is employed to represent macro influencers, “I” is utilized to indicate micro influencers, and “R” is utilized to denote regular users. For the third digit, “a” corresponds to activists, “c” pertains to celebrities, “j” signifies journalists, “m” designates media entities, “o” represents organizations, “p” denotes politicians, and “g” is employed for grassroots. CO stands to coherency score, STL refers to the significant time lags.

Furthermore, we conducted calculations to identify the top two significant time lags (STL) between the highly co-evolved word pairs within each cluster, with time measured in days. If only one number is presented under STL, it indicates the presence of a single significant time lag. A value of 0 denotes no time lag. In Figure 1 and Table 6, the prefixes assigned to nodes (i.e., Me, Ma, Mi, and R) represent the language patterns used by mega, macro, micro influencers, and regular users, respectively.

Of particular note is the encoding system used for mentions, employing a four-digit format. The first digit corresponds to the author type. The second digit reflects the type of mentioned Twitter handle. The third digit signifies the profession of the mentioned Twitter handles. Finally, the fourth digit denotes the sequence of a specific mention. Detailed explanation concerning the semantic implications of these alphabetical characters is provided in the footnotes of Table 6. For example, “R_E-j-1” represents a regular user's mention of the first mega journalist.

The largest cluster, denoted as red, prominently revolves around macro influencers' utilization of the hashtag #domesticterrorists, which exhibits a strong co-evolution with the discourses of regular users and micro influencers pertaining to #violenceagainstwomenact, #nativeamericans, and #hispaniclivesmatter. Notably, these discussions within the red cluster also encompass negative sentiments concerning fear and death. Intriguingly, macro influencers' implementation of the hashtag #domesticterrorists preceded regular users' adoption of #hispaniclivesmatter by approximately 1 day and preceded their usage of the sentiment word fear by 3 days.

The second largest cluster, denoted by the color blue, revolved around the mentions made by regular users regarding a mega influencer (i.e., E-j-1) who authored the earliest tweet concerning solidarity between African and Asian Americans (See sample tweet from Mega 2 in Table 2). Mega influencers' mentions of the Twitter handles and hashtags supporting the idea of solidarity (e.g., #solidarity) as well as their use of the positive sentiment word *love*, were one to 3 days earlier than regular users' mentions of a mega influencer who tweeted first on solidarity. Regular users' mentions of this mega influencer were 1–2 days earlier than micro influencers' discourse around #fightracism and #blackandgold. The interconnectedness of this blue cluster, pertaining to the theme of solidarity, exhibited significant co-evolution with the mentions of another mega influencer (identified as Mega 1 in Table 2). This particular mega influencer criticized both the Black Lives Matter and Stop Asian Hate movements, emphasizing that Black individuals constituted the primary perpetrators of violence against both African and Asian Americans.

The cluster with the third highest magnitude (denoted by the color green) revolved around the utilization of the hashtag #palestinianlivesmatter by micro influencers. It was observed that micro influencers mentioned another micro influencer (i.e., I-g-1) who employed co-hashtags to combat racism and show solidarity with individuals of all ethnicities, particularly people of color. Notably, these mentions occurred 1–2 days prior to the employment of the hashtag #palestinianlivesmatter by micro influencers. This cluster pertaining to #palestinianlivesmatter exhibited a strong co-evolution with the mention of #endsystemicracism by regular users, with a 1-day time lag. Additionally, it co-evolved with regular users' mention of another regular user (i.e., R-g-1) who employed co-hashtags to advocate for supporting the lives of Palestinians on the same day.

The fourth largest cluster, denoted by the color purple, demonstrated a highly synchronized co-evolution of nodes occurring on the same day. Within this cluster, the co-evolution of discourse primarily involved micro influencers, mega influencers, and regular users, focusing on combating racism. Notably, the mentions and hashtags within this cluster predominantly originated from a micro influencer's tweet. The micro influencer (i.e., I-j-1) held the role of a syndicated political cartoonist and journalist and shared a cartoon highlighting the deterioration of humanity in America.

The cluster occupying the fifth largest position (designated as orange) revolved around the utilization of the hashtag #stopaapihate by regular users. This cluster exhibited a significant co-evolution with the mentions made by macro and micro influencers, specifically referring to a particular micro influencer (i.e., I-p-1) who was an Asian-American Congresswoman and an online platform dedicated to documenting Antifa and the far left (i.e., I-o-1), all of which occurred on the same day. Although the employment of the hashtag #stopaapihate by regular users took place approximately 6 days subsequent to the usage of the positive term “solidarity” by macro influencers, it preceded the adoption of the same positive term by micro influencers and regular users by ~1 day.

The sixth largest cluster, depicted in light blue, displayed a robust co-evolution of nodes primarily driven by regular users' utilization of the hashtag #naomiosaka. This hashtag was employed as a means to express support for the human rights of the an Afro-Asian tennis player Naomi Osaka. Notably, regular users' mentions of #naomiosaka occurred ~1 day prior to mega influencers' mention of #naomiosaka and a journalist (i.e., E-j-1) who were also endorsing and advocating for Naomi Osaka.

The seventh largest cluster, depicted in light green, showcased a close co-evolution of nodes primarily driven by macro influencers' utilization of the hashtag #pride for the celebration of Pride Month, which is dedicated to the LGBTQ community. Mega influencers and regular users demonstrated a similar co-evolution pattern by employing LGBTQ-related hashtags, such as #pride and #logo30, as well as mentioning a specific LGBTQ-related media (i.e., I-m-1) related to LGBTQ matters. Notably, the usage of these LGBTQ-related hashtags and mentions by mega influencers and regular users occurred ~1 day prior to macro influencers' adoption of the hashtag #pride.

The eighth largest cluster (denoted as gray 1) captured the co-evolution of discourses surrounding crime involving all four user types. For instance, macro influencers' mentions of a specific macro influencer (i.e., A-j-1) who brought attention to criminal behaviors among African-Americans and violence against Asian-Americans, occurred ~1 day prior to the mentions made by mega influencers. The two smaller clusters (gray 2 and gray 3) primarily exhibited co-evolutions related to far-left hashtags among macro influencers, as well as the evolving usage of the hashtag #domesticterroism between regular users and micro influencers.

#### 3.6.7. Solidarity as a broker bridging the diverse social media discourses

Figure 2 is another visualization of the coherency network. The difference between Figures 1, 2 is that the size of nodes is determined by their betweenness centrality. We have rescaled the node size to highlight key nodes with the highest betweenness centralities, which also act as bridges in the coherency network. This emphasis enables us to identify the most influential nodes within the network, influencing information flow between different clusters. Table 7 presents the top ten nodes exhibiting the highest betweenness centralities within the coherency network, along with the temporal gaps observed in the usage of identical terms across various categories of Twitter users. In Table 7, the column values corresponding to ME, MA, MI, and R represent the time lags observed among identical words within each type of user category (ME—mega, MA—macro, MI—micro, R—regular). The symbol “-” indicates that the particular word is not utilized by the related type of users. Positive values in the column denote time lags in advance, while negative values indicate time lags afterwards. A value of 0 signifies the absence of any time lags. Drawing insights from Figure 2 and Table 7, it becomes evident that the concept of solidarity assumes paramount significance as a pivotal node facilitating connections between diverse topics arising from the discourses involving co-hashtags. The hashtag #solidarity, employed by regular users and micro influencers, occupies a pivotal position at the intersection of the red and purple clusters, establishing links between anti-racism discussions and the discourse on anti-domestic terrorism. Furthermore, the word solidarity, as employed by mega influencers, serves to connect the green cluster revolving around #palestinianlivesmatter with the blue cluster that facilitates both the promotion and critique of solidarity. In terms of temporal dynamics, the analysis of time lags reveals that mega influencers' mention of #solidarity precedes that of micro influencers by 1 day and that of regular users by 2 days. Additionally, mega influencers' incorporation of solidarity into their discourse transpires ~6 days earlier compared to the usage patterns observed among the remaining three user types.

**Table 7:** Top 10 nodes with the biggest betweenness centralities in the coherency network and the times lags among the same words between different types of users.

**Node**	**Betweenness centrality**	**ME**	**MA**	**MI**	**R**
R#solidarity	0.0884	2	–	1	0
Ma#stopracism	0.0863	–	0	−2	−5
Mi#stopasianhatecrimes	0.0799	–	0	0	0
Me_solidarity	0.0750	0	−6	−6	−6
R#naomiosaka	0.0589	−1	–	–	0
Mi#solidarity	0.0463	1	–	0	−1
R#stopasianhatecrimes	0.0408	–	0	0	0
Ma#indigenous	0.0328	–	0	1	6
R#thinkbigsundaywithmarsha	0.0326	–	0	7	0
R_discrimination	0.0318	–	0	0	0

In addition to the concept of solidarity, the hashtags pertaining to the Stop Asian Hate movement demonstrated a significant role in establishing connections between diverse discourses on Twitter. Notably, the hashtag #stopasianhatecrimes, employed by both micro influencers and regular users, occupied strategic positions at the intersections of the red cluster and the orange cluster. This facilitated the bridging of discussions concerning the Stop Asian Hate movement with those revolving around the topic of anti-domestic terrorism.

Regular users' mention of the hashtag #naomiosaka served as a linkage between the light blue cluster focused on supporting Naomi Osaka, who is an Afro-Asian tennis player, and the blue cluster centered on the promotion of solidarity. By employing this hashtag, regular users facilitated the integration of discussions regarding support for Naomi Osaka with the broader discourse surrounding the promotion of solidarity.

## 4. Discussion

### 4.1. Case study implication: complex co-evolutions on the topic of cross-racial solidarity

The findings of this case study have important implications for the field of social media research, shedding light on the dynamics of discourse co-evolution and the role of coherency network analysis in understanding the connection between two racial justice movements and the creation of cross-racial solidarity (Moon, 2021). By utilizing coherency network analysis, this study provides a comprehensive view of the interactions and influences among different types of Twitter users, highlighting the significance of this methodological approach in uncovering the complexities of online discourse.

One key implication is the influence exerted by mega influencers, despite their small number, over the other three types of users. These influential figures played a pivotal role in stimulating larger-scale social media discussions on cross-racial solidarity by expressing contrasting opinions. Their use of co-hashtags such as #solidarity and the term “solidarity” triggered public discussions, with micro influencers acting as mediators between mega influencers and regular users. However, it is worth noting that regular users emerged as the most significant contributors for promoting public discussions on the topic of cross-racial solidarity.

The centrality analysis of the coherency network further underscores the importance of regular users in bridging the diverse topics that emerged from Twitter discourses using co-hashtags. Through their mention of specific influencers and their use of #solidarity, regular users acted as bridges between different discourse clusters, facilitating the coalescence of various perspectives and promoting a broader understanding of cross-racial solidarity. The time lag analysis also suggests that regular users' tweets might primarily impact the discourse of mega and micro influencers, highlighting the dynamic interactions and influence flow within the coherency networks.

Additionally, this study reveals that the discourses of macro influencers did not strongly co-evolve with those of mega influencers. However, macro influencers played a crucial role in promoting support for a wider range of racial movements, such as Hispanic Lives Matter, by emphasizing the issue of domestic violence. Furthermore, micro influencers contributed to the expansion of Twitter discourses on racial movements by leading the discourse on promoting #palestinianlivesmatter. The posts from regular users also demonstrated how other underrepresented communities, such as the LGBTQ community, utilized the co-hashtags to enhance their visibility within these discussions.

Overall, these findings highlight the importance of coherency network analysis in uncovering the intricate dynamics of discourse co-evolution and the influential role of different user types within social media platforms. By employing this methodological approach, researchers can gain a more comprehensive understanding of online discourse and its potential to foster cross-racial solidarity and promote visibility for underrepresented communities.

### 4.2. Methodology contributions: analyzing social media discourse dynamics through coherency network analysis

The methodological contribution of this study lies in the utilization of coherency network analysis to extend and enrich existing research on social media discourse. In contrast to prior studies that predominantly focused on social media texts, this study considers the interplay between social media users and the discourses that arise from their interactions (Wang and Zhou, 2021). By employing coherency network analysis, the study adopts a comprehensive approach that captures the intricate dynamics of social media conversations, encompassing both the individual posts and the collective exchanges between users. This methodological choice provides a holistic understanding of social media discourse, recognizing its inherently collective nature.

The employed coherency network analysis technique enables an examination of the most frequently mentions, hashtags, and sentiment words used by four distinct categories of Twitter users. Through this analysis, the study identifies prominent and relevant topics within the discourses. Additionally, the analysis uncovers the influencers associated with these topics by leveraging the co-hashtags. This methodological approach offers valuable insights into the structure and dynamics of social media discourse. It highlights key individuals or entities driving conversations and exerting influence within specific thematic areas. By focusing on mentions, hashtags, and sentiment words, this study captures not only the textual content but also the social relationships and emotional nuances embedded within the discourse, thus providing a comprehensive depiction of social media interactions.

In conjunction with content analysis, the study conducts a time lag analysis to investigate temporal relationships between nodes within the coherency network, aiming to reveal potential causal connections. By examining the time delays between the emergence of certain discourses and subsequent responses or reactions from other users or influencers, the study provides insights into the directionality and influence flow within social media conversations. This methodological approach enhances our understanding of the multi-level interactions between influencers and regular users, shedding light on how different social media groups engage with and respond to each other's ideas. Consequently, it yields a nuanced comprehension of the co-creation of meanings in online environments.

By employing coherency network analysis, the study extends the scope of research beyond individual texts, delving into the intricate dynamics of user interactions and resulting discourses. This comprehensive approach enhances our understanding of the collective nature of social media conversations. Furthermore, the analysis of co-hashtags and influencers provides insights into the salient topics and influential figures driving these conversations. The time lag analysis further illuminates the causal relationships and directionality within the coherency network, deepening our understanding of how different social media groups collaborate and exert influences on each other. Overall, these methodological contributions allow us to better comprehend the co-creation of meanings and online social dynamics.

## 5. Limitation and future research

This study employs a novel approach, namely coherency network analysis, to investigate the bridging of two distinct racial justice movements through the use of co-hashtags (#BlackLivesMatter and #StopAsianHate). However, it is essential to acknowledge the limitations inherent in this research, which, in turn, present opportunities for future scholarly exploration in this domain.

To extend the current understanding, future investigations could apply the coherency network analysis approach to diverse contexts and conduct additional case studies. By examining a broader range of social media discourses and interactions, researchers can gain a more nuanced comprehension of how diverse hashtags are used across various topics and movements.

In the present study, user categorization is operationalized based on follower size, serving as a proxy for influence or reach. However, to enhance the sophistication of user classification, future research could explore alternative categorization methods. This may involve differentiating between organizational and individual users, considering their field of expertise, offline reputation, or employing other relevant factors, such as demographics related to political ideology, gender, race, and ethnicity. Such refined categorization strategies would facilitate a more in-depth exploration of the roles played by different user types in shaping social media discourse.

Furthermore, the time lag analysis conducted in this study employed a daily unit of analysis. However, to gain a more granular understanding of discourse co-evolution among various actors on Twitter, future investigations could adopt smaller time intervals, such as minutes or even seconds, as the unit of analysis. This enhanced temporal resolution would provide a more detailed examination of the temporal relationships between nodes within the coherency network, offering valuable insights into the dynamics of information dissemination and interaction on social media platforms.

In addition to the current research endeavors in coherency network analysis of social media text, there are also numerous promising directions for future investigations. One such avenue is conducting influence diffusion analysis on the coherency network, which can unveil the dynamics of information propagation and how ideas spread through interconnected nodes. Considering the inherent complexity and multifaceted nature of social media data, multilayer analysis presents an intriguing prospect. By integrating multiple layers of information, such as textual content, user interactions, and temporal aspects, researchers can gain a more comprehensive understanding of coherency networks.

In conclusion, future research endeavors can build upon these limitations by exploring diverse contexts, employing alternative user categorization methodologies, utilizing finer-grained time intervals, and incorporating more holistic approach for analysis. By doing so, scholars can advance our understanding of social media dynamics and discourse co-evolution, enriching the scholarly discourse in this field.

## Data availability statement

The raw data supporting the conclusions of this article will be made available by the authors, without undue reservation.

## Ethics statement

Ethical approval was not required for the study involving human data in accordance with the local legislation and institutional requirements. The social media data was accessed and analyzed in accordance with the platforms' terms of use and all relevant institutional/national regulations.

## Author contributions

KJ contributed to conception and design of the study, as well as the statistical analysis. QX wrote the literature review and part of the discussion. All authors contributed to manuscript revision, read, and approved the submitted version.
